# The Late Dr. F. R. Payne, of Marshall

**Published:** 1874-08-15

**Authors:** 


THE LATE DR. F. R. PAYNE, OF MARSHALL.—At the semi-annual meeting of the "Æsculapian Society of the Wabash Valley," held at Marshall, Illinois, May 27th and 28th, 1874, on motion of Dr. J. M. McKown, the following committee was appointed, viz.: W. M. Chambers, John Tenbrook and D. O. McCord, to draft resolutions expressing the feelings of the Society in reference to Dr. F. R. Payne, who died at his home in Marshall, December 2, 1873, of pneumonia, after an illness of one week, in the 53d year of his age.

The committee made the following report:

WHEREAS, It has been so ordered by the Allwise Disposer of life and death, that since the last annual meeting of this Society, our justly distinguished brother, Fleming Rich Payne, M.D., of Marshall, Illinois, should be removed from his earthly career of usefulness and intense mental and physical labor to that state of existence where reign perpetual peace and repose, where sickness and anguish have no abiding place; and

WHEREAS, His life was morally blameless, and his connection with this Society shed a lustre upon the organization; therefore,

*Resolved*, That by the death of Dr. Payne this Society has lost one of its oldest and best members; the medical literature of the State one of its ablest contributors; the profession at large the teaching and example of a thoroughly scientific physician and a most worthy Christian gentleman.

*Resolved*, That a copy of this preamble and resolutions be presented to the members of his greatly bereaved family by the Secretary, that they be entered upon the records of the Society and published in the MEDICAL EXAMINER, of Chicago.

---

ZOSTER FRONTALIS—*Treatment by the Galvanic Current, by A. D. Rockwell.*—A lady aged about sixty, and sent to me by Dr. C. R. Agnew, had suffered long and severely from zoster of the forehead and face. Acute and persistent neuralgia supervened, resisting all attempts at permanent alleviation. The galvanic current was locally and centrally applied, and resulted, in a few seances, in relieving in a good measure the neuralgic pains. Ptosis of the right eyelid remained, however, in spite of the treatment by galvanization. Three local applications of the faradic current approximately restored the lost muscular power.—*Phila. Med. Times.*

